# A new approach for obtaining rapid uniformity in rice (*Oryza sativa* L*.*) via a 3x x 2x cross

**DOI:** 10.1590/S1415-47572010005000023

**Published:** 2010-06-01

**Authors:** Shaochen Xing, Yuhong Cai, Kaida Zhou

**Affiliations:** 1 Biotechnology Research Center, Jilin Academy of Agricultural Sciences, ChangchunChina; 2 Research Center of Agricultural Environment and Resources, Jilin Academy of Agricultural Sciences, ChangchunChina; 3 Rice Research Institute, Sichuan Agricultural University, ChengduChina

**Keywords:** F-test, polyploidy, rice, SSR marker, stability

## Abstract

A triploid (2n = 3x = 36) rice plant was obtained by screening a twin seedling population in which each seed germinated to two or three sprouts that were then crossed with diploid plants. One diploid plant was chosen among the various F_1_ progenies and developed into an F _2_ population via self-pollination. Compared with the control variety Shanyou 63, this F _2_ population had a stable agronomical performance in field trials, as confirmed by the F-test. The stability of the F _2_ population was further substantiated by molecular analysis with simple sequence repeat markers. Specifically, of 160 markers assayed, 37 (covering all 12 chromosomes) were polymorphic between the parental lines. Testing the F _1_ hybrid individually with these markers showed that each PCR product had only a single band instead of two bands from each parent. The bands were identical to either maternal (23 markers) or paternal (eight markers) bands or distinct from both parents (six markers). The amplified bands of all 60 randomly selected F _2_ plants were uniform and identical to those of the F _1_ hybrid. These results suggest that the F _1_ plant is a non-segregating hybrid and that a stable F _2_ population was obtained. This novel system provides an efficient means for shortening the cycle of hybrid rice seed production.

Since the discovery that tetraploid plants can be regenerated from callus tissue on cut stems of diploid *Solanum nigrum* (Winkler, 1916) polyploidy has been recognized as a common phenomenon in nature and an important factor in the evolution of plant genomes. Polyploidy occurs in many taxa and is particularly widespread in flowering plants. At least half of the known angiosperm species have experienced polyploidy in their evolutionary history (Hieter and Griffiths, 1999; Echardt, 2001; Wu *et al.*, 2001). Polyploidy often results in considerable genomic changes such as chromosomal rearrangements, gene loss and changes in DNA methylation (reviewed by Adams, 2007).

Compared to their diploid and haploid counterparts, polyploid organisms often express specific characteristics such as larger cell and body sizes (Sugiyama, 2005) and a propensity to develop apomixis (Naumova *et al.*, 1999). Studies in rice have identified stable lines in an early generation from the progeny of 3x x 2x or 4x x 2x crosses (Wu *et al.*, 1999; Xing *et al.*, 2000). Wang *et al.* (1999) also reported that loss of heterozygosity (LOH) from 2x x 2x crosses led to stable panicle rows in F_2_ progeny and subsequently proposed a mechanism of “assortment mitosis” (Wang *et al.*, 2001) that was supported by cytological evidence (Wang *et al.*, 2006).

In this study, we screened another triploid x diploid cross that differs from the crosses reported by Wu *et al.* (1999) and obtained a diploid F_1_ plant that generated a stable F_2_ population. This system will be helpful in providing new insights into the potential application of polyploidy and should allow the development of an efficient breeding system to greatly shorten the breeding cycle.

Individuals of the triploid plant DB43, originally derived from a twin seedling population, served as the maternal parent. A diploid *japonica*-type cultivar, ZD2, served as the paternal parent. The 25 F_1_ seeds from a DB43/ZD2 cross were obtained by direct hybridization followed by embryo rescue. Five plants among the F_1_ seedlings were cytologically confirmed to be diploid (Xing and Zhou, 2000). Self-pollinated F_2_ seeds were collected to generate five F_2_ populations in the following year. Only one of these five populations appeared to be phenotypically uniform in the field.

To verify the phenotypic uniformity of the F_2_ population, five major morphological traits (plant height, panicle length, number of productive tillers, seed-setting rate and 1000-grain weight) were investigated and compared with the very widespread Shanyou 63 as the control variety by using the F-test (Table 1). The F value (sd_1_/sd_2_) for each trait was < 1.0, indicating that the F_2_ population was stable for these agronomical traits under the field conditions used.

Microsatellite markers were used to assess the relationship between parents and the F_1_ hybrid and to test the stability of the F_2_ population. PCR was done with the following assay mixture in 25 μL: 40 ng of template DNA, 200 μM of each of the four dNTPs, 2.5 μL of 10x buffer, 1 unit of DNA *Taq* polymerase, 2 mM MgCl_2_ and 0.25 μM of each of the two primers. The PCR amplifications were done in a Perkin Elmer 9600 GeneAmp PCR System with the following conditions: 94 °C for 7 min, followed by 35 cycles of 94 °C for 1 min, 55 °C for 1 min and 72 °C for 2 min, and a final extension at 72 °C for 10 min. The amplification products were separated by electrophoresis in 3% (w/v) agarose gels followed by staining with ethidium bromide and examination under UV light.

One hundred and sixty simple sequence repeat (SSR) markers were used to screen for polymorphisms in the parental lines: 37 of these markers covering all 12 rice chromosomes were polymorphic (Figure 1). More importantly, when these polymorphic markers were used to amplify the F_1_ DNA template individually, each F_1_ product showed only a single band instead of the expected two bands that were supposed to be identical to those from the two parents. Comparison of the PCR patterns of the parents with those of the F_1_ hybrid plant allowed the polymorphic SSR markers to be classified into three groups: Group 1 included 23 SSR markers for which the size of the band amplified from F_1_ was identical to that of the maternal parent (Figure 2A), Group 2 included eight SSR markers for which the size of the band amplified from F_1_ was identical to that of the paternal parent (Figure 2B) and Group 3 included six SSR markers for which the size of the F_1_ amplified band was completely different from either parent (Figure 2C). The 31 SSR markers in Groups 1 and 2 originated from either the maternal or paternal parent, rather than from both parents, implying that these loci are truly homozygous.

To confirm the uniformity of the F_2_ population, 60 DNA samples were randomly selected from the F_2_ population, together with DNA from both parents and the F_1_ hybrid, and used as templates for PCR amplification. The resulting PCR products from all of the polymorphic SSR markers were compared to each other on the same agarose gel. The resulting pattern indicated that all of the 60 samples were uniform and coincided with the genotype of F_1_ plant. Three markers representing each of the different groups and 30 F_2_ samples were chosen to illustrate this uniformity (Figure 2D).

Six markers had completely different PCR patterns with F_1_ DNA template from those of their parents. This phenomenon has also been observed in wheat (Liu *et al.*, 1998), although the mechanism of allele loss following hybridization remains unclear.

Various studies have shown that polyploidy can lead to immediate, extensive changes at the genic and genomic levels, resulting in differential gene silencing or gene loss (reviewed by Udall and Wendel, 2006). Josefsson *et al.* (2006) showed that maternal imprinting of PHERES1(PHE1), the gene of type I MADS-box, and paternal imprinting of MEDEA(MEA), the gene encodes a polycomb group (PcG) protein, appeared to be lost in hybrids between tetraploid *Arabidopsis thaliana* and diploid *Arabidopsisarenosa*. This phenomenon, known as early generation stability, has previously been reported in rice from apomixis (Chen, 1992), although not all studies have confirmed this (Shi *et al.*, 1996). The results of our experiment cannot be explained by apomixis because the markers tested in non-segregating diploid progeny were of mixed paternal and maternal origins. The most probable explanation in this case was recombination followed by chromosomal elimination in mitotic cells of the F_1_ hybrid.

Our results indicate that the F_2_ population was non-segregating and should theoretically be stable in subsequent generations. This unusual phenomenon, which differs from the findings previously reported by Wang *et al.* (1999), should prove useful for breeding restorer lines of hybrid rice (Zhou *et al.*, 2007).

**Figure 1:**
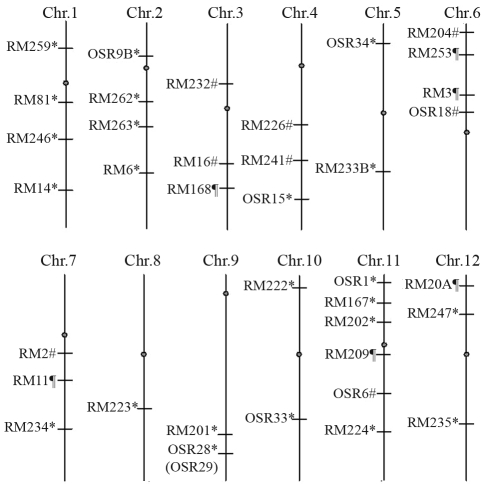
Chromosomal distribution of 37 polymorphic SSR markers among rice parental lines. The approximate positions of the markers and centromeres are based on the available genetic linkage maps for rice (Akagi *et al.*, 1996; Chen *et al.*, 1997; Temnykh *et al.*, 2000, 2001. The superscripts indicate three different groups and the dots indicate the positions of centromeres. *Group 1 markers for which the size of the amplified F_1_ band was identical to that of maternal band. ^#^Group 2 markers for which the size of the amplified F_1_ band was identical to that of paternal band. ^¶^Group 3 markers for which the size of the amplified F_1_ band was distinct from that of both parents.

**Figure 2:**
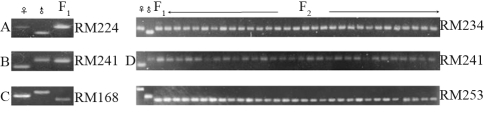
Pattern of PCR amplification for parental plants, F_1_ hybrid and F_2_ population. (A) RM224 marker in group 1: the size of the amplified F_1_ band was the same as that of the maternal plant. (B) RM241 marker in group 2: the size of the amplified F_1_ band was the same as that of the paternal plant. (C) RM168 marker in group 3: the size of the amplified F_1_ band was different from that of both parental plants. (D) Non-segregating amplified bands from F_2_ plants that were identical to amplified F_1_ bands, as assessed by using the markers RM234, RM 241 and RM 253 from groups 1, 2 and 3, respectively.

## Figures and Tables

**Table 1:** Comparison of stability for major agronomical traits between the F_2_ population and the control variety Shanyou 63 using the F-test.

Traits	F_2_ population	Control (Shaoyou 63)	F value
Plant height (cm)	125.9 ± 3.48	121.1 ± 6.32	0.55
Panicle length (cm)	24.7 ± 1.14	25.2 ± 1.53	0.75
Tiller number	8.6 ± 2.54	8.1 ± 2.9	0.87
Seed-setting rate (%)	60.7 ± 7.10	78.6 ± 10.1	0.70
1000-grain weight (g)	24.1 ± 1.03	28.4 ± 1.11	0.93

The values are the mean ± SD.
